# Differential open chromatin profile and transcriptomic signature define depot-specific human subcutaneous preadipocytes: primary outcomes

**DOI:** 10.1186/s13148-018-0582-0

**Published:** 2018-11-26

**Authors:** Adeline Divoux, Katalin Sandor, Dora Bojcsuk, Amlan Talukder, Xiaoman Li, Balint L. Balint, Timothy F. Osborne, Steven R. Smith

**Affiliations:** 1 Translational Research Institute for Metabolism and Diabetes, Florida Hospital, 301 E. Princeton Street, Orlando, FL 32804 USA; 2 Diabetes and Obesity Research Center, Sanford Burnham Prebys Medical Discovery Institute at Lake Nona, Orlando, FL USA; 3 Genomic Medicine and Bioinformatic Core Facility, Department of Biochemistry and Molecular Biology, Faculty of Medicine, University of Debrecen, Nagyerdei krt 98, Debrecen, 4032 Hungary; 4 Department of Computer Science, University of Central Florida, 4000 Central Florida Blvd, Orlando, 32816 USA; 5 Burnett School of Biomedical Sciences, College of Medicine, University of Central Florida, 6900 Lake Nona Boulevard, Orlando, FL USA; 6 Present address: Department of Medicine, Johns Hopkins All Children’s Hospital, Johns Hopkins University School of Medicine, St. Petersburg, FL 33701 USA

**Keywords:** Chromatin openness, Gene expression, Histone marks, Preadipocytes, Fat distribution, Abdominal fat, Gluteofemoral fat

## Abstract

**Background:**

Increased lower body fat is associated with reduced cardiometabolic risk. The molecular basis for depot-specific differences in gluteofemoral (GF) compared with abdominal (A) subcutaneous adipocyte function is poorly understood. In the current report, we used a combination of Assay for Transposase-Accessible Chromatin followed by sequencing (ATAC-seq), RNA-seq, and chromatin immunoprecipitation (ChIP)-qPCR analyses that provide evidence that depot-specific gene expression patterns are associated with differential epigenetic chromatin signatures.

**Methods:**

Preadipocytes cultured from A and GF adipose tissue obtained from premenopausal apple*-*shaped women were used to perform transcriptome analysis by RNA-seq and assess accessible chromatin regions by ATAC-seq. We measured mRNA expression and performed ChIP-qPCR experiments for histone modifications of active (H3K4me3) and repressed chromatin (H3K27me3) regions respectively on the promoter regions of differentially expressed genes.

**Results:**

RNA-seq experiments revealed an A-fat and GF-fat selective gene expression signature, with 126 genes upregulated in abdominal preadipocytes and 90 genes upregulated in GF cells. ATAC-seq identified almost 10-times more A-specific chromatin-accessible regions. Using a combined analysis of ATAC-seq and global gene expression data, we identified 74 of the 126 abdominal-specific genes (59%) with A-specific accessible chromatin sites within 200 kb of the transcription start site (TSS), including HOXA3, HOXA5, IL8, IL1b, and IL6. Interestingly, only 14 of the 90 GF-specific genes (15%) had GF-specific accessible chromatin sites within 200 kb of the corresponding TSS, including HOXC13 and HOTAIR, whereas 25 of them (28%) had abdominal-specific accessible chromatin sites. ChIP-qPCR experiments confirmed that the active H3K4me3 chromatin mark was significantly enriched at the promoter regions of HOXA5 and HOXA3 genes in abdominal preadipocytes, while H3K27me3 was less abundant relative to chromatin from GF. This is consistent with their A-fat specific gene expression pattern. Conversely, analysis of the promoter regions of the GF specific HOTAIR and HOXC13 genes exhibited high H3K4me3 and low H3K27me3 levels in GF chromatin compared to A chromatin.

**Conclusions:**

Global transcriptome and open chromatin analyses of depot-specific preadipocytes identified their gene expression signature and differential open chromatin profile. Interestingly, A-fat-specific open chromatin regions can be observed in the proximity of GF-fat genes, but not vice versa.

**Trial registration:**

Clinicaltrials.gov, NCT01745471. Registered 5 December 2012.

**Electronic supplementary material:**

The online version of this article (10.1186/s13148-018-0582-0) contains supplementary material, which is available to authorized users.

## Introduction

As body weight increases, the distribution of body fat becomes an important factor that determines metabolic health [1]. Excess intra-abdominal fat storage is well-known to be associated with increased cardiovascular and metabolic risks [2, 3]. As compared to other fat depots, visceral adipose tissue has increased metabolic activity, specifically both lipid uptake and lipolysis. Free fatty acids, the product of lipolysis, induce peripheral insulin resistance thereby inhibiting skeletal muscle uptake. However, over the past 15 years, growing evidence supports a primary role of subcutaneous adipose tissue in the metabolic complications of obesity and in the development of insulin resistance [4–7]. Importantly, Jensen demonstrated that the upper body non-visceral fat was the major source of free fatty acids in humans, with intra-abdominal (visceral) adipose tissue contributing to only 15% of the total systemic free fatty acids [6]. A very recent study where the authors collected the data from the National Health and Nutrition Examination Survey for the years 1999–2006 established in the same manner a strong association between upper body fat and metabolic risk factors independent of visceral fat [8]. In contrast, preferential fat accumulation in the lower body, or gluteal-femoral (GF) depot, is now recognized to be protective against the deleterious cardiometabolic effects of obesity [9, 10]. A common hypothesis is that lower body fat serves as a “sink” for excess energy sequestering lipid away from visceral adipose tissue and also preventing fat accumulation in other unhealthy areas such as the liver, pancreas, and muscle [11]. Regional gene expression differences that influence preadipocyte proliferation, differentiation, and subtype abundance likely contribute to regional variations in fat tissue function (for reviews, see [12–14]). However, the exact physiological, metabolic, or endocrine mechanisms underlying the beneficial effects of lower body fat deposition remain unknown.

Multiple studies, including our own work, reported that the homeobox (HOX) family and HOX domain-encoding genes (SHOX2 and IRBX2) are differentially expressed between the two major subcutaneous tissue depots, abdominal and GF [15–17]. Interestingly, preadipocytes isolated from these depots, cultured and differentiated under the same conditions, retain the unique gene expression profile characteristic of depot origin, and the differences are maintained during conversion into lipid-laden adipocytes [18, 19]. Importantly, we discovered that the lncRNA HOTAIR known to be involved in the epigenetic regulation of cancer progression [20, 21] is only expressed at appreciable levels in GF-fat and isolated preadipocytes from GF [22]. Altogether, these observations strongly indicate there is a depot-specific epigenetic program that is involved in adipose tissue patterning which may be also influence pathologic downstream effects in obesity.

Current knowledge is limited concerning the chromatin status of adipose tissue from different anatomical locations. To date, only one study investigated the DNA methylome of the human A- and GF-fat by using DNA methylation array in a heterogenous group of subjects [15]. Chromatin accessibility represents another essential level of genome regulation. In the present study, we investigated depot-specific chromatin structure and gene expression using preadipocytes isolated from human subcutaneous adipose tissue. We report on the differences in chromatin openness and transcriptomic signatures of the A and GF preadipocytes in order to better understand the basis for the differential programming of anatomically distinct scWAT depots.

## Methods

### Participants

Six healthy premenopausal, weight-stable females aged 20–40 years with a body mass index (BMI) (calculated as weight in kilograms divided by height in meters squared) between 27 and 40 kg/m^2^ were recruited in Orlando using advertisements approved by an institutional review board. Subjects were excluded if they reported a history of chronic disease (diabetes, heart or liver disease, high blood pressure, gastrointestinal disorder), recent weight loss or gain (> 3 kg over the past 8 weeks), had abnormal blood or urine values, or use of oral contraceptives or hormone replacement therapy. Adipose tissue biopsies were collected with a three-hole 2.5-mm liposuction cannula from the mid-abdomen approximately 5–8 cm lateral to the umbilicus. Gluteofemoral adipose tissue was collected 10–20 cm below the greater trochanter on the most lateral side of the upper thigh. Samples were cleaned at the bedside; a fraction of it was snap frozen in liquid nitrogen, and the rest was immediately used for preadipocyte isolation.

The characteristics of the study group are presented in Table 1. Two subjects of this group were randomly selected to perform genome-wide analysis.

**Table 1:** Clinical parameters of women subjects (mean ± standard deviation)

Clinical parameters	Obese subjects (*n* = 6)
Age (years)	34 ± 4.7
Adiposity markers	
BMI (kg/m^2^)	34.0 ± 2.81
Body weight (kg)	95.4 ± 6.80
Waist to hip ratio	0.94 ± 0.04
Total fat mass (kg)	40.6 ± 4.73
Total lean mass (kg)	53.2 ± 3.92
% fat mass	43.0 ± 2.92
Metabolic markers	
Systolic blood pressure (mm Hg)	113 ± 4.85
Diastolic blood pressure (mm Hg)	68.0 ± 6.02
Glucose (mg/dL)	94.5 ± 12.9
Insulin (mU/L)	6.24 ± 2.85
FFA (mmol/L)	0.43 ± 0.06
TSH (mUI/L)	1.59 ± 0.36

### Isolation of preadipocytes and culture conditions

Stromal vascular fractions were isolated from scWAT abdominal and GF depot, plated, and grown as previously described [23] with the addition of hEGF and hFGF (Life Technologies, 10 μg/ml and 4 μg/ml, respectively) during the expansion phase [24]. The preadipocyte population was initially purified by depletion of the stromal vascular fraction from macrophages (CD14- and CD206-positive cells) and endothelial cells (CD31-positive cells) using fluorescence-activated cell sorting. At confluence, cells were harvested and used for Assay for Transposase-Accessible Chromatin followed by sequencing (ATAC-seq) or chromatin isolation as described above. A fraction of the cells were frozen for RNA extraction.

### RNA-seq

RNA was isolated using RNeasy Mini Kit (Qiagen) from abdominal and GF preadipocytes. Approximately 2 μg was used for library preparation with TruSeq RNA Sample Preparation Kit (Illumina). Following purification, RNA was fragmented with divalent cations at 85 °C, and then cDNA was generated by random primers and SuperScript II enzyme (Life Technologies). Second-strand synthesis was performed followed by end repair, single “A” base addition, and ligation of barcode indexed adaptors to the DNA fragments. Adapter-specific PCRs were performed to generate sequencing libraries. Libraries were size selected with E-Gel EX 2% agarose gels (Life Technologies) and purified by QIAquick Gel Extraction Kit (Qiagen). The result is libraries with inserts ranging in size from 120 to 210 bp with a median size of 155 bp. Libraries were sequenced on HiSeq 2500 instrument.

### ATAC-seq

ATAC-seq was carried out as described earlier [25]. After trypsinization, abdominal and GF preadipocytes nuclei were isolated. Nuclei were used for tagmentation using Nextera DNA Library Preparation Kit (Illumina). After tagmentation, DNA was purified with MinElute PCR Purification Kit (Qiagen). Tagmented DNA was amplified with Kapa HiFi Hot Start Kit (Kapa Biosystems) using 16 PCR cycles. Amplified libraries were purified with Agencourt AMPure XP (Beckman Coulter). Fragment distribution of libraries was assessed with Agilent Bioanalyzer, and libraries were sequenced on a HiSeq 2500 platform.

### Real-time quantitative RT-PCR

Total RNA from adipose tissue and preadipocytes were extracted using the lipid RNeasy kit and the RNeasy mini kit (Qiagen), respectively. Gene expression was assessed by real-time PCR using a ViiA7 sequence detection system (Life Technologies) and Taqman technology suitable for relative gene expression quantification using the following parameters: 1 cycle of 95 °C for 10 min, followed by 40 cycles at 95 °C for 10 s and 60 °C for 1 min. All samples were normalized to PPIA showing low variability between A and GF samples (Additional file 1: Figure S1).

### Chromatin Immunoprecipitation-PCR assay

Chromatin immunoprecipitations (ChIPs) were performed and analyzed as described [26]. Briefly, cells were crosslinked for 10 min with 1% formaldehyde and quenched 8 min with glycine (125 mM). Cells were incubated in buffer A (10 mM Tris, 1 mM KCl, 1.5 mM MgCl2) for 10 min on ice then dounce homogenized with 10 strokes. The mixture was centrifuged, and the pellet was resuspended in buffer B (50 mM Tris-HCl, 10 mM EDTA, 1% SDS) and kept for 10 min on ice. DNA was sonicated to obtain 300–1000 bp fragments with the Diagenode Bioruptor sonicator (high setting with 30 s on/off cycle during 15 min). The rabbit polyclonal H3K4me3 and H3K27me3 antibodies (Diagenode) were used to study the histone marks; a Rabbit IgG was used as a negative control. Dynabeads (Life Technologies) blocked with PBS 1% BSA were used to immunoprecipitate the complexes.

ChIP-PCR primers (Table 2) were designed to amplify the promoter regions from the specific HOX genes as detailed in Figs. 5 and 6. Primers were also designed for a region of the GAPDH locus for a positive control and G6Pase for a negative control.

**Table 2:** List of primer sequences used for real-time PCR to quantify the ChIP-assays

Gene	Forward	Reverse
HOTAIR (−329)	GTGAGCTCGCGGCATTTTTA	GCATTTTCGACCCAGGCATC
HOXA3 (− 313)	GGGGGTAGGGAGGAATTTGC	CCTGACCCCCAAGAACTCAC
HOXA5 (− 398)	TGGCTAAATGGCTTTCCCCC	CCGTTTTGCAGCCCCTCTTA
HOXC13 (+ 42)	CCAATCCAGAGACTTCAGGA	GAGGAGAGCGCTGTAACTG

### RNA-seq analysis

Raw sequence data were aligned to the hg19 reference genome assembly (GRCh37) by using TopHat (v2.0.7). The fragments per kilobase of transcript per million mapped reads (FPKM) values were calculated by Cufflinks (v2.0.2) with default parameters [27]. Differentially expressed genes per subjects were determined by the Cuffdiff algorithm of Cufflinks, and only genes with *p* ≤ 0.05 in both subjects were used to further analyses (Additional file 2: Table S1). Gene expression heat map was visualized by using the *pheatmap* function of the pheatmap package in R. The values were normalized per rows.

### ATAC-seq analysis

Raw sequence reads were aligned to the hg19 reference genome assembly (GRCh37) by using the Burrows-Wheeler Alignment (BWA) tool (v07.10) [28], and then BAM files were generated with SAMtools (v0.1.19) [29]. “Peaks” were predicted using the Model-based Analysis of ChIP-Seq (MACS2) tool (v2.0.10) [30]. To remove the artifacts from the predicted peaks, we used the blacklisted genomic regions of the Encyclopedia of DNA Elements (ENCODE) [31]. Fragment length was set to 150 nucleotides by the *makeTagDirectory* program of the Hypergeometric Optimization of Motif EnRichment (HOMER) package (v4.2) [32].

Coverage (bedgraph) files were created by the *makeUCSCfile.pl* program of HOMER. A consensus set was generated from those peaks which could be predicted from at least two samples, and “summit coverage” values (on the middle 50-bp bins) were calculated based on this set by *annotatePeaks.pl* (HOMER) with *-hist 50* and *-ghist* parameters. Each value within the coverage files was divided with the median value of “summit coverage.” Upon this normalization, bedgraph files were converted to tiled data formats (.tdf) by using the *toTDF* command of the IGVtools program (v2.3.9) [33]. Median normalized read distribution plots of overlapping and depot-specific peaks were also generated. Peak annotation was determined by *annotatePeaks.pl*. Histograms, created also with *annotatePeaks.pl*, show the average tag densities of the peaks relative to the transcription start site (TSS) ± 250 bp in 1-bp resolution. Overlapping and depot- or subject-specific peaks were determined using the *intersectBed* program of BedTools (v2.23.0) [34].

### DiffBind analysis

Correlation heat maps of ATAC-seq peak densities were created by differential binding analysis (DBA) using the DiffBind package (v1.2.4) in R. Regions that were predicted from at least two samples (minOverlap parameter was set to 2) and were found differently opened between the depots according to a *P* ≤ 0.005 threshold were used for visualization and further analyses. For volcano plots, significance and log fold change values were calculated by DiffBind. ATAC-seq peaks within ± 100 kb to the TSS of depot-specific genes but not farther than the neighboring gene (BedTools) were used for further DiffBind analyses.

### Motif analysis

Motif enrichment analysis was carried out by *findMotifsGenome.pl* (HOMER). It was performed on the ± 100 bp flanking regions of the peak summits or in the ± 250 bp regions of genes TSSs. The search lengths of the motifs were 8, 10, 12, and 14 bp. *p* values were calculated by comparing the enrichments within the target regions and those of a random set of regions (background) generated by HOMER.

### Visualization of the ATAC-seq data

Volcano plots, box plots, and histograms were created with GraphPad Prism 6. Coverage files were visualized by Integrative Genomics Viewer (IGV).

### RT-qPCR and ChIP-qPCR analysis

To compare A-fat vs. GF-fat gene expression and histone marks in obese subjects, individual data are presented and paired *t* tests were used. ChIP-PCR data are presented as a percent of 5% input. All statistical analyses on clinical data were performed in SAS (V11.2.1).

## Results

### Characterization of the accessible chromatin profile of human subcutaneous preadipocytes

**Fig. 1:**
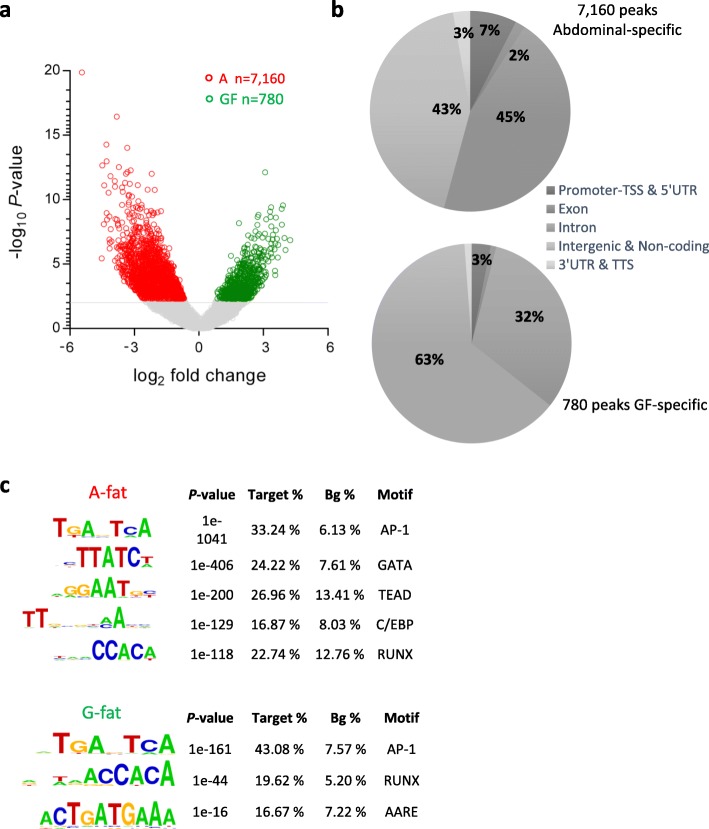
Depot-specific open chromatin regions identify specific transcription factor motifs. **a** Volcano plot representation of depot-specific open chromatin regions in preadipocytes. Abdominal- (highlighted in red) and GF-specific (highlighted in green) open chromatin regions are presented (*p* < 0.005). **b** Genomic distribution of the depot-specific open chromatin regions. **c** Motif analysis of the depot-specific open chromatin regions. Enriched motif matrices are presented along with the *p* values; the percentages of each motif are found in the target (Target %) and background (Bg %) genomic regions

We set out to study the open chromatin profile of human subcutaneous white adipose tissue (WAT), using abdominal (A) and gluteofemoral (GF) depot-derived preadipocytes isolated from two obese apple-shaped women (age = 33 ± 2.3 years, BMI = 34 ± 1.4 kg/m^2^, waist to hip ratio = 0.93 ± 0.02). In order to gain a genome-wide view of accessible chromatin regions from the isolated preadipocytes, we utilized Assay for Transposase-Accessible Chromatin followed by sequencing (ATAC-seq). We sequenced two technical replicates from each individual’s A- and GF-derived preadipocytes yielding four samples from each depot. We identified 112,362 and 104,957 peaks in A and GF samples respectively, 91,022 peaks being common to both depots (81% of the A peaks and 87% of the GF peaks). Differential binding analysis of the ATAC-seq data and hierarchical clustering revealed that A and GF samples are clustered together as expected based on their distinct anatomical origin (Additional file 3: Figure S1). Further analysis of the ATAC-seq data identified the significantly different open chromatin regions between A and GF preadipocytes. We observed 7160 A-specific and 780 GF-specific open chromatin regions (Fig. 1a and Additional file 3: Figure S1). Genome browser visualizations of the top 5 A- and GF-specific regions in each sample were summarized in supplemental data (Additional file 3: Figure S2).

Genome-wide distribution of depot-specific accessible chromatin regions revealed that around half of these were localized in intergenic regions (43% of A-specific regions and 63% of GF-specific regions) as shown in Fig. 1b. In addition, a higher percentage of abdominal-specific regions were found in gene promoter regions (7%) compared to the GF-specific peaks (only 3%) (Fig. 1b). De novo motif analysis at these specific open chromatin regions identified some of the well-known transcription factors involved in adipogenesis, such as GATA and C/EBP (Fig. 1c). As expected, a clear majority of the depot-specific peaks showed enrichment for the transcription factor binding motif AP-1, which is known to promote WAT formation (Fig. 1c).

### Gene expression profiling of human subcutaneous preadipocytes

**Fig. 2:**
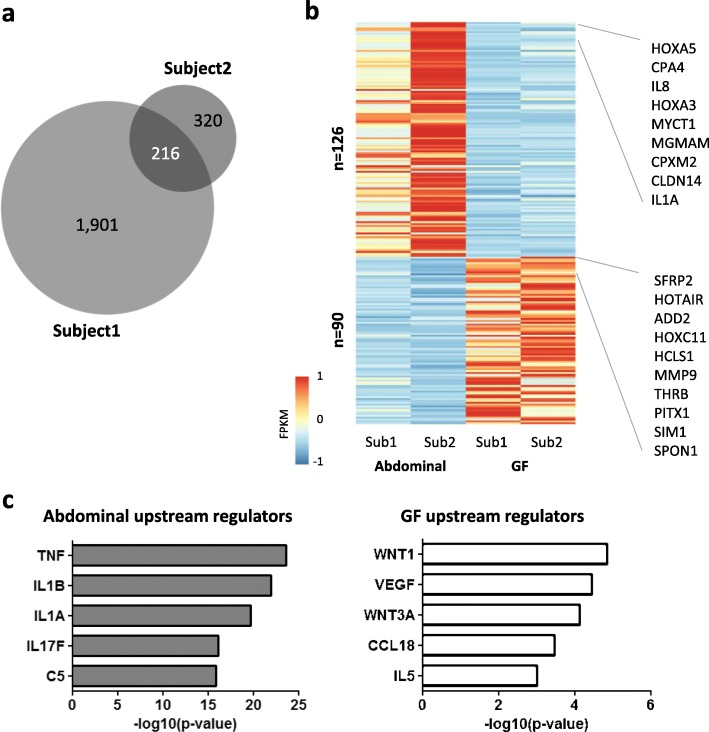
Depot-specific gene expression in preadipocytes. **a** Venn diagram depicts the number of genes that were differentially expressed between abdominal and GF preadipocytes in each subject or in both (*p* < 0.05). **b** Heat map representation of depot-specific gene expression determined by RNA-seq from the abdominal and GF depots of two subjects (Sub). The top 10 signature genes of each depot are listed. **c** Ingenuity Pathway Analysis of the potential upstream regulators of abdominal- and GF-specific gene sets. Upstream regulators that fall into the category of cytokines and growth factors are presented

After we determined the accessible chromatin regions of depot-specific preadipocytes, we investigated the associated gene expression profiles. In the first subject, 2117 genes were differentially expressed between abdominal and GF preadipocytes. The difference was much smaller for the second subject with only 536 differentially expressed genes (Fig. 2a). In this study, we have focused on the 216 genes that were differentially regulated in both subjects. Of these, 126 genes exhibited significantly higher expression in abdominal preadipocytes, and 90 genes showed significantly higher expression in GF preadipocytes (Fig. 2b, Additional file 2: Table S1). Consistent with our previously described results from adipose tissue [16], some of the HOX gene family members showed the greatest fold change difference in our datasets between the two depots (HOXA5 and HOXA3 in the abdominal depot—fold change 86 and 26, respectively; HOXC11 in GF depot—fold change 24). Interestingly, inflammatory cytokines (IL1α, IL1β) and chemokines (IL8, GCP2, GRO1/2/3, CXCL5, MCP1) were upregulated in abdominal preadipocytes whereas expression of extracellular matrix components (SFRP2, ADD2, MMP9, SPON1, HTRA3, CDON) were found preferentially increased in GF cells. We performed Ingenuity Pathway Analysis (IPA) to gain further insight into the possible functional implications of these gene expression signatures. Potential upstream regulators of the depot-specific gene sets show that abdominal-specific genes are highly likely to be regulated by inflammatory cytokines (TNF, IL1B, IL1A, IL17F, and C5) than GF-specific genes; however, chemokines were observed as the upstream regulators of both gene groups (Fig. 2c). GF-specific genes were enriched for the activators of the WNT-signaling pathway consistent with decreased adipose expansion for the GF depot in apple-shaped women (Fig. 2c).

### Integration of transcriptional and chromatin openness data

**Fig. 3:**
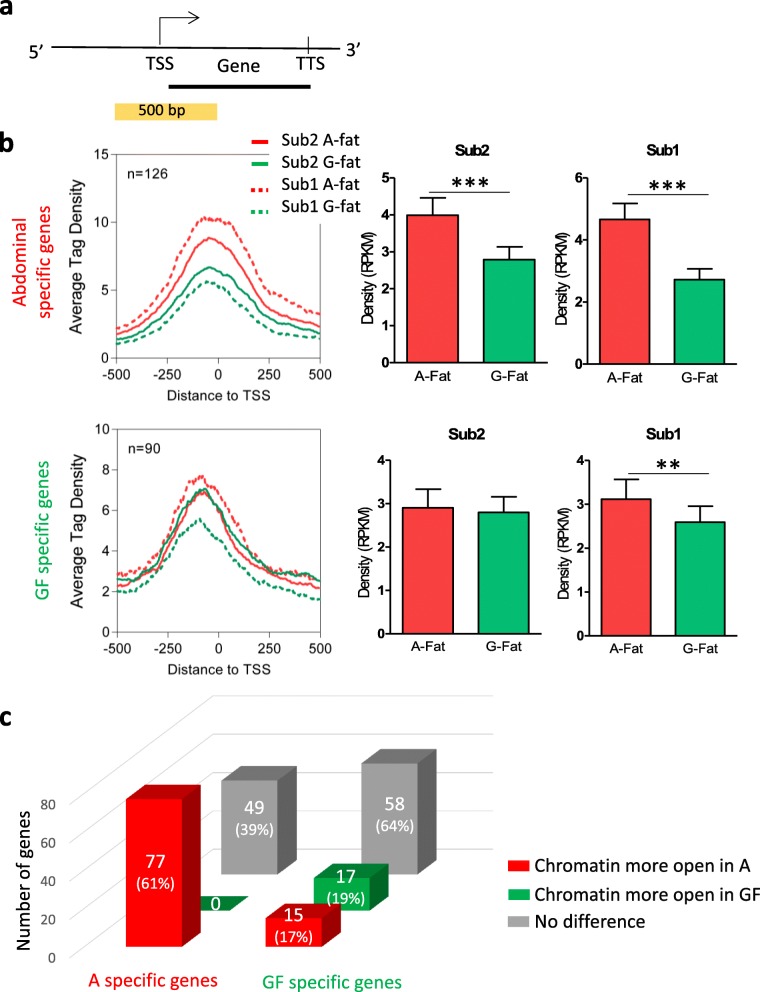
Promoter accessibility of depot-specific genes. **a** Schematic representation of the investigation of promoter accessibility using ATAC-seq. ATAC-seq signals were calculated around the transcription start sites (TSSs) of the depot-specific genes in a 500-bp-wide window (250 bp up- (5′) and downstream (3′) relative to the TSSs). **b** Histogram-based depiction of the average tag density at the promoter regions of abdominal- (*n* = 126) and GF-specific (*n* = 90) genes in a 1-kb window centered on the TSSs. ATAC-seq density around the TSS of abdominal and GF depot-specific genes in each depot and for each subject (DiffBind). Sub1 = Subject 1, Sub2 = Subject 2 paired *t* test; **p < 0.01, ***p < 0.005. **c** The number of genes with open promoter in abdominal (red) or GF (green) depot, according to their expression specificity (on the left side are the genes upregulated in abdominal preadipocytes, on the right side are the genes upregulated in GF preadipocytes). The gray bars represent the genes with no differential opened chromatin between A and GF depot

To examine the correlation between depot-specific open chromatin regions from the ATAC-seq analysis with depot-specific gene expression signatures, we integrated our ATAC-seq and RNA-seq datasets. We first focused on the ATAC-seq signals at the proximal promoter that we defined as a region covering 250 bp upstream and downstream from the transcriptional start site (TSS) of the differentially expressed genes (Fig. 3a). Normalized read enrichments at the promoter regions of the 126 abdominal-specific genes reported significantly higher chromatin accessibility in the abdominal depot compared to the GF depot, for both subjects (Fig. 3b). Importantly, the same analysis on the gene promoters of the 90 GF signature genes did not show major differences, although we observed significantly less reads from the GF preadipocytes of subject 2 (Fig. 3b).

To provide a much more detailed view on chromatin openness on the abdominal- and GF-specific gene promoters, we calculated the ratio of the RPKM values of the ATAC-seq data from the two depots on each promoter for genes that showed depot-specific expression. This analysis allowed us to define more accessible promoter regions using a 1.5-fold change cutoff. Interestingly, more than 39% of abdominal- and close to 65% of GF-specific genes promoters did not follow the expression of the corresponding gene with regard to their chromatin openness (Fig. 3c). As expected, 61% of the abdominal specific-genes promoters exhibited more accessibility in abdominal, and none of the abdominal-specific genes showed more open chromatin in GF cells (Fig. 3c). Surprisingly, the GF-specific gene promoters showed a mixed open chromatin profile between the depots, 19% being more open in GF cells and 17% being more open in abdominal cells (Fig. 3c).

**Fig. 4:**
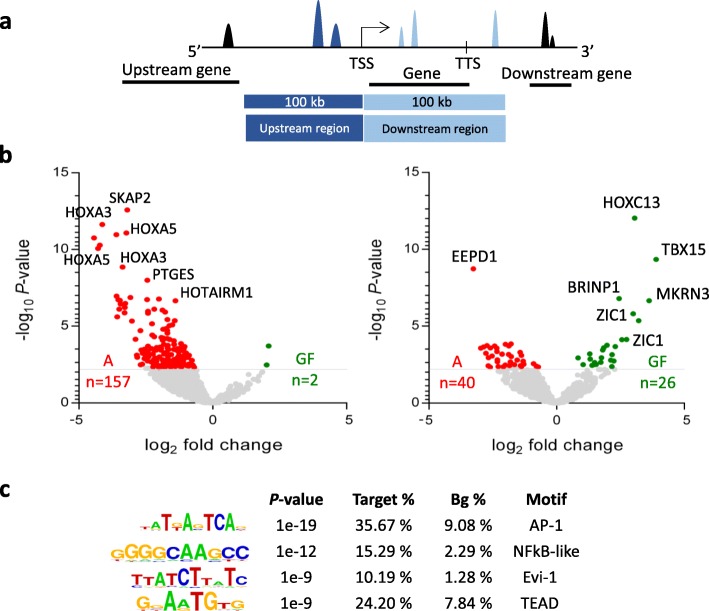
Identification of the putative regulatory regions of depot-specific genes. **a** Depot-specific open chromatin regions determined by ATAC-seq were annotated to the nearest depot-specific genes in a ± 100-kb genomic region relative to the transcription start sites (TSSs) and (transcription termination site (TTS)). **b** Volcano plots portray the depot-specific open chromatin regions annotated to the abdominal- (left graph) and GF-specific genes (right graph). Abdominal-specific open chromatin regions are highlighted in red, while GF-specific open chromatin regions are highlighted in green. **c** Motif analysis of the 159 abdominal-specific open chromatin regions annotated to abdominal specific genes. Enriched motif matrices are presented along with the *p* values; the percentages of each motif are found in the target (Target%) and background (Bg%) genomic regions

To further investigate the relationship between chromatin openness and gene expression, we decided to extend our analysis to a broader genomic region around the TSSs of the depot-specific genes. Therefore, we annotated depot-specific open chromatin regions that are located in a genomic region of 100 kbs up- and downstream of the TSSs of the depot-specific genes (Fig. 4a). Our analysis identified 157 abdominal dominant open chromatin regions in the proximity of the abdominal-specific genes that are linked to 74 differentially expressed genes based on our annotation criteria (Fig. 4b left panel and Additional file 2: Table S2). The two well-established markers of abdominal depot HOXA5 and HOXA3 exhibited abdominal-specific expression and could be linked to seven and five abdominal-specific open chromatin regions, respectively. As expected, only two GF-specific regions could be annotated to abdominal genes (Fig. 4b left panel and Additional file 2: Table S2). We observed a total of 66 GF-dominant open chromatin regions in the proximity of the GF-dominant genes, linked to 39 unique genes (Fig. 4b right panel and Additional file 2: Table S2). Surprisingly, 25 of these genes had significantly more open chromatin regions in the abdominal depot (corresponding to 40 ATAC-seq peaks), and only 14 genes had more open chromatin regions in the GF depot (corresponding to 26 ATAC-seq peaks) (Fig. 4b right panel). The already reported GF-specific HOXC13 gene possessed one of the most reliably detected GF-specific open chromatin regions.

After annotating the depot-dominant/specific open chromatin regions to the depot-specific genes, we next carried out de novo motif analysis on the 157 abdominal-specific open chromatin regions. Our results revealed transcription factor motifs involved in the regulation of inflammation (AP-1, NFĸB-like motif) (Fig. 4c). Motif analysis on the abdominal-dominant open chromatin regions near GF-specific genes did not reveal significantly enriched motifs compared to the background, which can be explained by the low number [35] of target regions included in this analysis; however, the motifs found may function as response elements of transcriptional repressors (Additional file 3: Figure S3).

### Active and repressive histone modifications positively correlate with promoter openness and gene expression of depot-specific genes

**Fig. 5:**
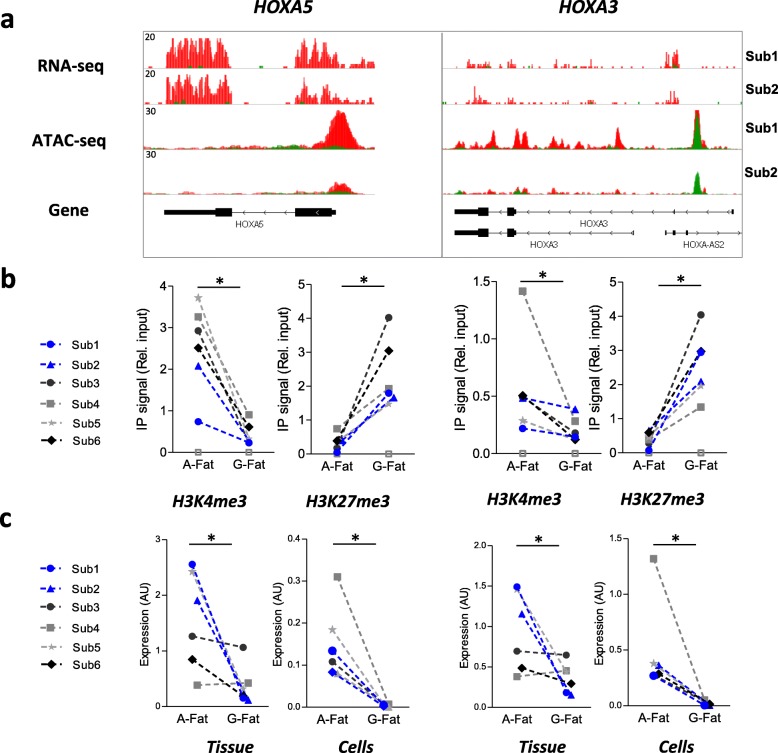
Validation of Abd-specific gene expression and its correlation with active and repressive histone marks. **a** IGV genome browser view of RNA-seq and ATAC-seq signals on the indicated loci from abdominal (red) and GF (green) preadipocytes. Overlay tracks are presented for each gene loci. **b** ChIP-qPCR assay in abdominal and GF preadipocytes with H3K4me3 antibody and H3K27me3 antibody at the promoter region of the selected genes. **c** RT-qPCR assay for HOXA5 and HOXA3 in abdominal and GF preadipocytes. Wilcoxon test *p < 0.05. White squares represent the IgG negative control

**Fig. 6:**
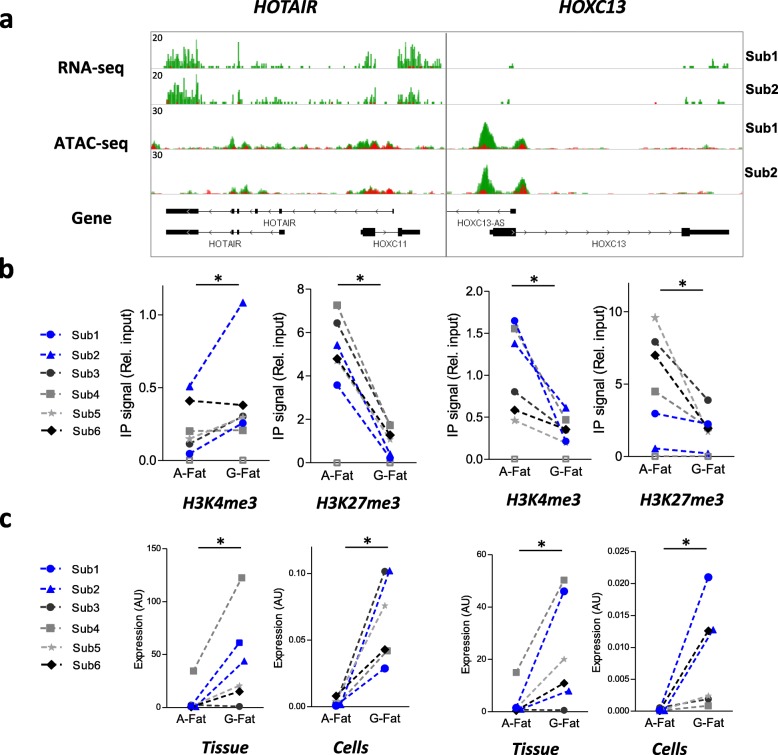
Validation of GF-specific gene expression and its correlation with active and repressive histone marks. **a** IGV genome browser view of RNA-seq and ATAC-seq signals on the indicated loci from abdominal (red) and GF (green) preadipocytes. Overlay tracks are presented for each gene loci. **b** ChIP-qPCR assay in abdominal and GF preadipocytes with H3K4me3 antibody and H3K27me3 antibody at the promoter region of the selected genes. **c** RT-qPCR assay for HOXC13 and HOTAIR in abdominal and GF preadipocytes. Wilcoxon test *p < 0.05. White squares represent the IgG negative control

To confirm the validity of the data obtained by genome-wide sequencing technologies on these two subjects, we selected four depot-specific genes (abdominal-specific: HOXA5, HOXA3; GF-specific: HOXC13, HOTAIR) (Figs. 5a and 6a), isolated preadipocytes on a larger cohort of subjects (*n* = 6 obese, apple-shaped women, including the two subjects previously studied), and carried out promoter-based ChIP-qPCR for a select group of histone modifications known to be associated with active (H3K4me3) or repressed (H3K27me3) chromatin. We also performed RT-qPCR on the whole tissue and on the preadipocytes to measure the mRNA levels of the depot-specific genes. ChIP-qPCR experiments confirmed that H3K4me3 was significantly enriched at the promoter regions of HOXA5 and HOXA3 genes in abdominal preadipocytes, while H3K27me3 was less abundant (Fig. 5b). Conversely, analysis of the promoter regions of the GF-specific HOTAIR and HOXC13 genes exhibited high H3K4me3 and low H3K27me3 levels compared to abdominal preadipocytes (Fig. 6b). mRNAs of the HOXA5 and HOXA3 genes were consistent with the RNA-seq datasets and showed significantly higher expression in the abdominal depot and cells (Fig. 5b), whereas HOXC13 and HOTAIR mRNA were expressed significantly higher in the GF depot and cells (Fig. 6b).

## Discussion

 A substantial number of studies have focused on identifying extrinsic regulators of preadipocytes and adipocytes, whereas comparatively very little is known about the chromatin structure differences that might control their identity and/or functions. A recent study showed the importance of chromatin modifications in the maintenance of the preadipocyte state by keeping the levels of the adipogenic master regulatory genes (PPARγ and C/EBPα) low. Similarly, high expression levels of these transcription factors upon adipocyte differentiation were also dependent on the presence of certain chromatin modifications [36], suggesting the importance of unique epigenetic signatures in the control of fat-specific gene expression. Global DNA methylomes of human scWAT have been related to BMI and body fat distribution [37], but only a small fraction of DNA methylation changes have been correlated to unique patterns of gene expression. However, this study did not take into consideration the cell composition of adipose tissue that can shift according to the degree of adiposity and inflammation. In our study, we investigated and compared the chromatin state of preadipocytes isolated from human abdominal and GF-fat and its correlation with cell gene expression signatures. Chromatin accessibility profiles of each depot were highly similar at a qualitative level, yet thousands of regions exhibiting quantitative differences in chromatin accessibility were also identified. Almost 8000 differentially accessible regions were observed between the depots. Using DNase hypersensitive site (DHS)-seq (technology comparable to ATAC-seq) during 3 T3-L1 adipogenesis, Mandrup’s group identified 22,383 novel DHS sites at the early phase of differentiation, the majority of which being transiently open [38]. At the end of the differentiation, only 11,936 new DHS sites were observed in mature adipocytes. In another study performed on human α- and β-cells previously sorted, Ackermann et al. revealed 27,000 β-cell-specific ATAC-seq peaks whereas only 1850 ATAC-seq peaks were identified as α-cell-specific [39]. In our study, we compared two kinds of subcutaneous preadipocytes, defined by their differential anatomical localization, culture in the exact same condition. “8000 hits” is in the range of the data previously described and seems a reasonable number. Additional experiments on human adipose tissue are however necessary to discuss more in detail the number of differentially open regions we found. Interestingly, chromatin isolated from abdominal preadipocytes showed ten times more ATAC-seq signals compared to the GF cells, suggesting a global increase of open chromatin in this depot. Given the fact that the two participants included in our analysis have a predominant accumulation of fat in their abdominal region, the specific chromatin signature observed in this depot may reflect changes in preadipocytes activity such as proliferation and differentiation into mature adipocytes, both occurring during adipose development. Arner et al. reported a similar pattern with higher global mean DNA methylation observed in obese women compared to never obese women [40]. In our study, motif analysis performed on the abdominal-specific open chromatin regions also highlighted transcription factors known to be involved in proliferation (TEAD), adipocyte differentiation (GATA) [35], and adipocyte commitment (AP-1) [41] (Fig. 1c), consistent with the previous literature. GF-specific ATAC-seq signals showed a surprising enrichment for amino acid response elements (AARE) binding sites, implying for the first time a role for amino acid signaling in scWAT distribution. Amino acid deficiency has been shown to induce endoplasmic reticulum (ER) stress, itself known to couple obesity to other metabolic dysfunctions [42, 43]. By extrapolation, we speculate an increase of ER stress in GF-fat that may lead to potential preadipocyte dysfunction, making preadipocytes unable to properly differentiate into mature adipocytes to sequester excess calories. These events could lead to abnormal fat accumulation in the upper body and the emergence of metabolic disorder. However, our actual study did not show any evidence of differential ER stress level between the two scWAT depots. It is important to note that the two subjects we analyzed here were obese but did not show further signs of metabolic dysfunction. To validate our hypothesis, it would be necessary to investigate in more detail the GF depot in subjects presenting comorbidities.

Using RNA-seq, we identified 126 genes upregulated in abdominal cells and 90 genes upregulated in GF cells. In accordance with our previous studies [16, 22], the fat depot specificity of some HOX genes and the lncRNA HOTAIR is maintained in isolated preadipocytes. Pathway analysis with ingenuity suggested an upregulation of inflammatory signaling in the abdominal depot, whereas the GF depot seems to be more affected by extracellular matrix remodeling-related signaling pathways, suggesting a depot-specific pattern. Future studies need to confirm these preliminary observations in a larger number of apple-shaped subjects and to also explore the transcriptional patterns of cells isolated from pear-shaped women.

The strong association observed between open chromatin and active gene expression in A-fat supports the role of differential chromatin signatures in the transcriptional regulation of abdominal-specific genes. Inversely, GF-specific genes displayed a loss in GF chromatin accessibility within 200 kb of the TSS, reflecting a more complex situation in lower body fat depots. Surprisingly, we observed abdominal-specific ATAC-seq signals around the TSS of 25 GF-specific genes, which may suggest the presence of active transcriptional repressors at these open chromatin regions. Transcription factor motif analysis confirmed the presence of the DNA-binding motif for the transcriptional repressors, NF1, and/or ZNF263 at these abdominal-dominant open chromatin regions. ATAC-seq regions identified in abdominal depot near the TSS of GF-specific genes were also enriched for H3K27me3, which is the marker of repressed chromatin territories (data not shown). Altogether, these observations suggest the presence of abdominal-specific repressive mechanisms that affect the chromatin landscape of GF-specific gene loci and gene expression signature, but further functional studies are required to provide evidence about the existence of such phenomenon.

As described in our previous study [22], we observed a dramatic upregulation of the linc HOTAIR in the obese GF depot compared to the abdominal depot (fold change 28 in tissue and cells—Fig. 6c). The role of HOTAIR is well described in cancer where HOTAIR acts as a modular scaffold and interacts directly with PRC2 and LSD1 complexes, recruiting them to target gene loci, and represses their transcription via H3K27-trimethylation (PRC2 complex and EZH2 activity) and H3K4-demethylation (LSD1 activity) (for review [44]). Interestingly, there is a strong support that these two methyltransferases EZH2 and LSD1 promote adipogenesis [45, 46]. Overall, our limited ChIP-qPCR analysis for H3K27 and H3K4me3 is consistent with the changes in PRC2 and LSD1 activity and suggests that HOTAIR may play a deterministic role in the association observed between gene expression and chromatin structure in scWAT that merits further investigation.

Several studies revealed adipose tissue depot-specific expression of multiple HOX genes [47] and their implication in metabolic diseases [48]. HOXA1, A4, and HOXC4 have been previously found directly involved in the differentiation and further generation of human white and brown adipose tissue, showing the importance of HOX genes at the early stages of adipocyte differentiation and commitment. In the present study, chromatin structure and histone marks of some of the HOX genes were associated with their gene expression in both scWAT depots (HOXA5, HOXA3, and HOXC13). Their expression pattern is also preserved between adipose tissue and cells (Figs. 5 and 6), suggesting an epigenetic mechanism for control of their transcription. This correlation may be a simple consequence of their differential anatomical origin; however, the robust HOX gene signature observed in tissue and cells remains intriguing, and the question of their involvement in fat development and distribution remains unresolved.

Regarding the limitations of our study, our genome-wide analysis was performed only on two apple-shaped women. Future studies must involve more subjects not only for validation purposes but also to extend our analysis and provide a more comprehensive view on the potential epigenomic programming of depot-specific adipocytes, especially on women presenting with a gynoid fat pattern. A previous analysis of adipogenesis regulator genes performed in human mesenchymal stem cells revealed dynamic changes in histone marks during the differentiation [49]. However, other studies have found that histone modifications were globally stable throughout differentiation and showed distinct and highly dynamic distribution patterns at specific genes, indicating that histone modifications in mesenchymal stem cells appear to be gene-specific [50]. Here, we investigated the chromatin and histone modifications only in preadipocytes isolated from scWAT depots, and thus, we did not address their stability through the process of differentiation. It would be important to carry out follow-up experiments in fully differentiated adipocytes as well as in adipocytes freshly isolated from abdominal and GF-fat tissue to more fully investigate epigenomic control of transcriptional regulation of scWAT depot-specific genes.

In conclusion, our analysis revealed that the open chromatin landscapes for abdominal vs. GF preadipocytes exhibit significant differences, suggesting depot-specific mechanisms that shape the cellular epigenome, according to their anatomical origin. In addition, the different open chromatin profile positively associates with a depot-specific gene expression signature, supporting a role for chromatin status in the regulation of the depot-selective patterns of scWAT gene expression. Finally, our gene-specific analyses show that our findings are reproducible with available technologies and highlight a strong positive correlation between chromatin openness and the presence of active and repressive chromatin marks in preadipocytes.

## Additional files

Additional file 1: Additional figure with data on PPIA gene expression. Representation of the individual PPIA gene Ct value obtained by RT-qPCR in cells (white squares) and tissue (black squares). A = abdominal depot, G = GF depot. (PPTX 51 kb)

Additional file 2: Additional tables with data on RNA-seq and ATAC-seq list of genes. **Table S1.** Differentially expressed genes between abdominal and GF depot in subject 1, subject 2, and in both. **Table S2.** List of open chromatin regions in the proximity of the depot-specific genes. (ZIP 231 kb)

Additional file 3: Additional figures with data on ATAC-seq. **Figure S1.** Differential binding analysis identifies depot-specific chromatin accessibility in preadipocytes. a) Correlation plot of accessible chromatin regions defined by ATAC-seq from different subjects (Sub1 and Sub2), fat depots (gluteofemoral-GF, abdominal-A), and technical replicates (rep#1 and rep#2). b) Identification of differentially open chromatin regions from GF and abdominal preadipocytes using differential binding analysis (DiffBind). Heat map representation of the abdominal- (*n* = 7160) and the GF-specific (*n* = 780) open chromatin regions. Sub = subject, Rep = technical replicate. The pairwise correlation scores were used for hierarchical clustering (*p* < 0.005, DiffBind). **Figure S2.** Depot-specific open chromatin regions of preadipocytes. IGV genome browser view of depot-specific ATAC-seq signals from abdominal (red) and GF (green) preadipocytes. Abdominal- (left) and GF-specific open chromatin regions are shown. Results are shown from two subjects and from 2-2 technical replicates. Chromosomal locations are indicated at the bottom for each genomic loci. Figure S3. Motif analysis on the 40 abdominal-specific open chromatin regions annotated to GF specific genes. Enriched motif matrices are presented along with the *p* values, the percentages of each motif found in the target (Target%) and background (Bg%) genomic regions. (PPTX 146 kb)

## Abbreviations

A-fat: Abdominal fat
ATAC: Assay for transposase-accessible chromatin
BMI: Body mass index
ChIP: Chromatin immunoprecipitation
FFA: Free fatty acid
FPKM: Fragments per kilobase of transcript per million mapped reads
GF-fat: Gluteofemoral fat
hEGF: Human epidermal growth factor
hFGF: Human fibroblast growth factor
HOX: Homeobox
IPA: Ingenuity Pathway Analysis
lncRNA: Long non-coding RNA
RPKM: Reads per kilobase of transcript per million mapped reads
scWAT: Subcutaneous white adipose tissue
TSS: Transcription start site
WHR: Waist to hip ratio

## References

[1] Vague J (1996). The degree of masculine differentiation of obesities: a factor determining predisposition to diabetes, atherosclerosis, gout, and uric calculous disease. 1956. Obes Res.

[2] Fontbonne A, Thibult N, Eschwege E, Ducimetiere P (1992). Body fat distribution and coronary heart disease mortality in subjects with impaired glucose tolerance or diabetes mellitus: the Paris Prospective Study, 15-year follow-up. Diabetologia.

[3] Pouliot MC, Despres JP, Nadeau A, Moorjani S, Prud’Homme D, Lupien PJ, Tremblay A, Bouchard C (1992). Visceral obesity in men. Associations with glucose tolerance, plasma insulin, and lipoprotein levels. Diabetes.

[4] Patel P, Abate N (2013). Role of subcutaneous adipose tissue in the pathogenesis of insulin resistance. J Obes.

[5] Fried SK, Lee MJ, Karastergiou K (2015). Shaping fat distribution: new insights into the molecular determinants of depot- and sex-dependent adipose biology. Obesity (Silver Spring).

[6] Jensen MD (2006). Is visceral fat involved in the pathogenesis of the metabolic syndrome? Human model. Obesity (Silver Spring).

[7] Jialal I, Devaraj S. Subcutaneous adipose tissue biology in metabolic syndrome. Horm Mol Biol Clin Invest. 2018;33(1):1868-91. 10.1515/hmbci-2017-0074 29353263

[8] Grundy SM, Williams C, Vega GL (2018). Upper body fat predicts metabolic syndrome similarly in men and women. Eur J Clin Investig.

[9] Pinnick KE, Nicholson G, Manolopoulos KN, McQuaid SE, Valet P, Frayn KN, Denton N, Min JL, Zondervan KT, Fleckner J (2014). Distinct developmental profile of lower-body adipose tissue defines resistance against obesity-associated metabolic complications. Diabetes.

[10] Yusuf S, Hawken S, Ounpuu S, Bautista L, Franzosi MG, Commerford P, Lang CC, Rumboldt Z, Onen CL, Lisheng L (2005). Obesity and the risk of myocardial infarction in 27,000 participants from 52 countries: a case-control study. Lancet.

[11] Tchkonia T, Thomou T, Zhu Y, Karagiannides I, Pothoulakis C, Jensen MD, Kirkland JL (2013). Mechanisms and metabolic implications of regional differences among fat depots. Cell Metab.

[12] Karpe F, Pinnick KE (2015). Biology of upper-body and lower-body adipose tissue--link to whole-body phenotypes. Nat Rev Endocrinol.

[13] Tchkonia T, Giorgadze N, Pirtskhalava T, Thomou T, DePonte M, Koo A, Forse RA, Chinnappan D, Martin-Ruiz C, von Zglinicki T (2006). Fat depot-specific characteristics are retained in strains derived from single human preadipocytes. Diabetes.

[14] Van Harmelen V, Rohrig K, Hauner H (2004). Comparison of proliferation and differentiation capacity of human adipocyte precursor cells from the omental and subcutaneous adipose tissue depot of obese subjects. Metab Clin Exp.

[15] Gehrke S, Brueckner B, Schepky A, Klein J, Iwen A, Bosch TC, Wenck H, Winnefeld M, Hagemann S (2013). Epigenetic regulation of depot-specific gene expression in adipose tissue. PLoS One.

[16] Karastergiou K, Fried SK, Xie H, Lee MJ, Divoux A, Rosencrantz MA, Chang RJ, Smith SR (2013). Distinct developmental signatures of human abdominal and gluteal subcutaneous adipose tissue depots. J Clin Endocrinol Metab.

[17] Passaro A, Miselli MA, Sanz JM, Dalla Nora E, Morieri ML, Colonna R, Pisot R, Zuliani G (2017). Gene expression regional differences in human subcutaneous adipose tissue. BMC Genomics.

[18] Hauner H, Entenmann G (1991). Regional variation of adipose differentiation in cultured stromal-vascular cells from the abdominal and femoral adipose tissue of obese women. Int J Obes.

[19] Tchoukalova YD, Koutsari C, Votruba SB, Tchkonia T, Giorgadze N, Thomou T, Kirkland JL, Jensen MD (2010). Sex- and depot-dependent differences in adipogenesis in normal-weight humans. Obesity (Silver Spring).

[20] Gupta RA, Shah N, Wang KC, Kim J, Horlings HM, Wong DJ, Tsai MC, Hung T, Argani P, Rinn JL (2010). Long non-coding RNA HOTAIR reprograms chromatin state to promote cancer metastasis. Nature.

[21] Tsai MC, Manor O, Wan Y, Mosammaparast N, Wang JK, Lan F, Shi Y, Segal E, Chang HY (2010). Long noncoding RNA as modular scaffold of histone modification complexes. Science (New York, NY).

[22] Divoux A, Karastergiou K, Xie H, Guo W, Perera RJ, Fried SK, Smith SR (2014). Identification of a novel lncRNA in gluteal adipose tissue and evidence for its positive effect on preadipocyte differentiation. Obesity (Silver Spring)..

[23] Lee MJ, Wu Y, Fried SK (2012). A modified protocol to maximize differentiation of human preadipocytes and improve metabolic phenotypes. Obesity (Silver Spring)..

[24] Hebert TL, Wu X, Yu G, Goh BC, Halvorsen YD, Wang Z, Moro C, Gimble JM (2009). Culture effects of epidermal growth factor (EGF) and basic fibroblast growth factor (bFGF) on cryopreserved human adipose-derived stromal/stem cell proliferation and adipogenesis. J Tissue Eng Regen Med.

[25] Buenrostro JD, Giresi PG, Zaba LC, Chang HY, Greenleaf WJ (2013). Transposition of native chromatin for fast and sensitive epigenomic profiling of open chromatin, DNA-binding proteins and nucleosome position. Nat Methods.

[26] Barish GD, Yu RT, Karunasiri MS, Becerra D, Kim J, Tseng TW, Tai LJ, Leblanc M, Diehl C, Cerchietti L (2012). The Bcl6-SMRT/NCoR cistrome represses inflammation to attenuate atherosclerosis. Cell Metab.

[27] Trapnell C, Williams BA, Pertea G, Mortazavi A, Kwan G, van Baren MJ, Salzberg SL, Wold BJ, Pachter L (2010). Transcript assembly and quantification by RNA-Seq reveals unannotated transcripts and isoform switching during cell differentiation. Nat Biotechnol.

[28] Li H, Durbin R (2009). Fast and accurate short read alignment with Burrows-Wheeler transform. Bioinformatics.

[29] Li H, Handsaker B, Wysoker A, Fennell T, Ruan J, Homer N, Marth G, Abecasis G, Durbin R (2009). The sequence alignment/map format and SAMtools. Bioinformatics.

[30] Zhang Y, Liu T, Meyer CA, Eeckhoute J, Johnson DS, Bernstein BE, Nusbaum C, Myers RM, Brown M, Li W (2008). Model-based analysis of ChIP-Seq (MACS). Genome Biol.

[31] Dunham I, et al. An integrated encyclopedia of DNA elements in the human genome. Nature. 2012;489(7414):57–74. 10.1038/nature11247 PMC3439153 22955616

[32] Heinz S, Benner C, Spann N, Bertolino E, Lin YC, Laslo P, Cheng JX, Murre C, Singh H, Glass CK (2010). Simple combinations of lineage-determining transcription factors prime cis-regulatory elements required for macrophage and B cell identities. Mol Cell.

[33] Thorvaldsdottir H, Robinson JT, Mesirov JP (2013). Integrative Genomics Viewer (IGV): high-performance genomics data visualization and exploration. Brief Bioinform.

[34] Quinlan AR, Hall IM (2010). BEDTools: a flexible suite of utilities for comparing genomic features. Bioinformatics.

[35] Tong Q, Dalgin G, Xu H, Ting CN, Leiden JM, Hotamisligil GS (2000). Function of GATA transcription factors in preadipocyte-adipocyte transition. Science.

[36] Matsumura Y, Nakaki R, Inagaki T, Yoshida A, Kano Y, Kimura H, Tanaka T, Tsutsumi S, Nakao M, Doi T (2015). H3K4/H3K9me3 bivalent chromatin domains targeted by lineage-specific DNA methylation pauses adipocyte differentiation. Molecular Cell.

[37] Agha G, Houseman EA, Kelsey KT, Eaton CB, Buka SL, Loucks EB (2015). Adiposity is associated with DNA methylation profile in adipose tissue. Int J Epidemiol.

[38] Siersbaek R, Nielsen R, John S, Sung MH, Baek S, Loft A, Hager GL, Mandrup S (2011). Extensive chromatin remodelling and establishment of transcription factor ‘hotspots’ during early adipogenesis. EMBO J.

[39] Ackermann AM, Wang Z, Schug J, Naji A, Kaestner KH (2016). Integration of ATAC-seq and RNA-seq identifies human alpha cell and beta cell signature genes. Mol Metab.

[40] Arner P, Sinha I, Thorell A, Ryden M, Dahlman-Wright K, Dahlman I. The epigenetic signature of subcutaneous fat cells is linked to altered expression of genes implicated in lipid metabolism in obese women. Clin Epigenetics. 2015;7:93. 10.1186/s13148-015-0126-9 PMC4562340 26351548

[41] Luther J, Ubieta K, Hannemann N, Jimenez M, Garcia M, Zech C, Schett G, Wagner EF, Bozec A (2014). Fra-2/AP-1 controls adipocyte differentiation and survival by regulating PPARgamma and hypoxia. Cell Death Differ.

[42] Lee J, Ozcan U (2014). Unfolded protein response signaling and metabolic diseases. J Biol Chem.

[43] Scheuner D, Kaufman RJ (2008). The unfolded protein response: a pathway that links insulin demand with beta-cell failure and diabetes. Endocr Rev.

[44] Bhan A, Mandal SS (2015). LncRNA HOTAIR: a master regulator of chromatin dynamics and cancer. Biochim Biophys Acta.

[45] Wei Y, Chen YH, Li LY, Lang J, Yeh SP, Shi B, Yang CC, Yang JY, Lin CY, Lai CC (2011). CDK1-dependent phosphorylation of EZH2 suppresses methylation of H3K27 and promotes osteogenic differentiation of human mesenchymal stem cells. Nat Cell Biol.

[46] Musri MM, Carmona MC, Hanzu FA, Kaliman P, Gomis R, Parrizas M (2010). Histone demethylase LSD1 regulates adipogenesis. J Biol Chem.

[47] Hilton C, Neville MJ, Karpe F (2013). MicroRNAs in adipose tissue: their role in adipogenesis and obesity. Int J Obes (2005).

[48] Procino A, Cillo C (2013). The HOX genes network in metabolic diseases. Cell Biol Int.

[49] Mikkelsen TS, Xu Z, Zhang X, Wang L, Gimble JM, Lander ES, Rosen ED (2010). Comparative epigenomic analysis of murine and human adipogenesis. Cell.

[50] Zhang Q, Ramlee MK, Brunmeir R, Villanueva CJ, Halperin D, Xu F (2012). Dynamic and distinct histone modifications modulate the expression of key adipogenesis regulatory genes. Cell Cycle.

