# Supplementary figures and images for: Differential open chromatin profile and transcriptomic signature define depot-specific human subcutaneous preadipocytes: primary outcomes
Source: Clin Epigenetics. 2018 Nov 26;10:148. doi: 10.1186/s13148-018-0582-0 (PMC6258289; doi:10.1186/s13148-018-0582-0)

## Slide 1
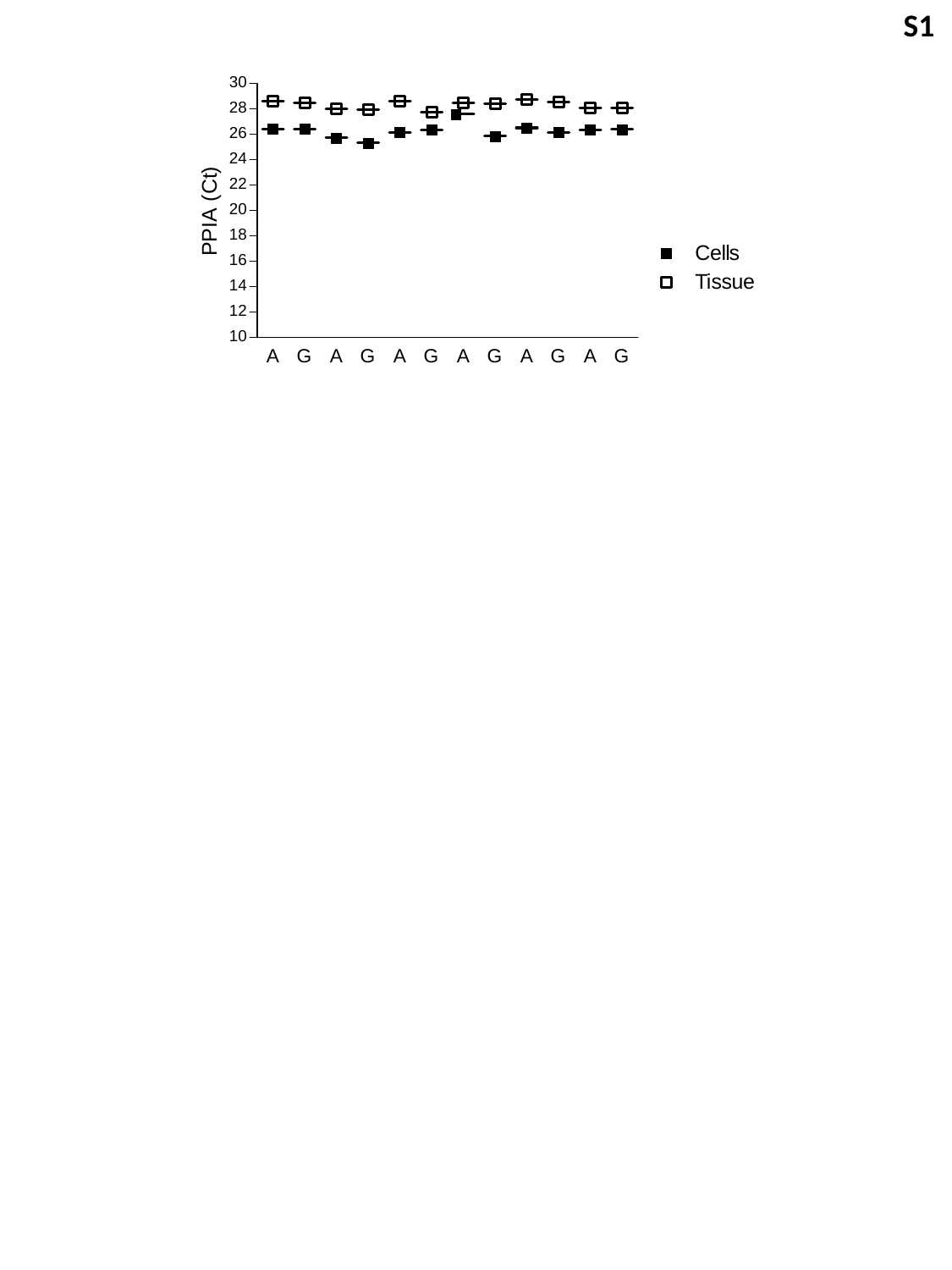

S1

Supplement: Supplementary file 1 — Additional figure with data on PPIA gene expression. Representation of the individual PPIA gene Ct value obtained by RT-qPCR in cells (white squares) and tissue (black squares). A = abdominal depot, G = GF depot. (PPTX 51 kb) [file 13148_2018_582_MOESM1_ESM.pptx]
