# Supplementary material for: Differential open chromatin profile and transcriptomic signature define depot-specific human subcutaneous preadipocytes: primary outcomes
Source: Clin Epigenetics. 2018 Nov 26;10:148. doi: 10.1186/s13148-018-0582-0 (PMC6258289; doi:10.1186/s13148-018-0582-0)
Supplement: Supplementary file 3 — Additional figures with data on ATAC-seq. Figure S1. Differential binding analysis identifies depot-specific chromatin accessibility in preadipocytes. a) Correlation plot of accessible chromatin regions defined by ATAC-seq from different subjects (Sub1 and Sub2), fat depots (gluteofemoral-GF, abdominal-A), and technical replicates (rep#1 and rep#2). b) Identification of differentially open chromatin regions from GF and abdominal preadipocytes using differential binding analysis (DiffBind). Heat map representation of the abdominal- (n = 7160) and the GF-specific (n = 780) open chromatin regions. Sub = subject, Rep = technical replicate. The pairwise correlation scores were used for hierarchical clustering (p < 0.005, DiffBind). Figure S2. Depot-specific open chromatin regions of preadipocytes. IGV genome browser view of depot-specific ATAC-seq signals from abdominal (red) and GF (green) preadipocytes. Abdominal- (left) and GF-specific open chromatin regions are shown. Results are shown from two subjects and from 2-2 technical replicates. Chromosomal locations are indicated at the bottom for each genomic loci. Figure S3. Motif analysis on the 40 abdominal-specific open chromatin regions annotated to GF specific genes. Enriched motif matrices are presented along with the p values, the percentages of each motif found in the target (Target%) and background (Bg%) genomic regions. (PPTX 146 kb) [file 13148_2018_582_MOESM3_ESM.pptx]

## Slide 1
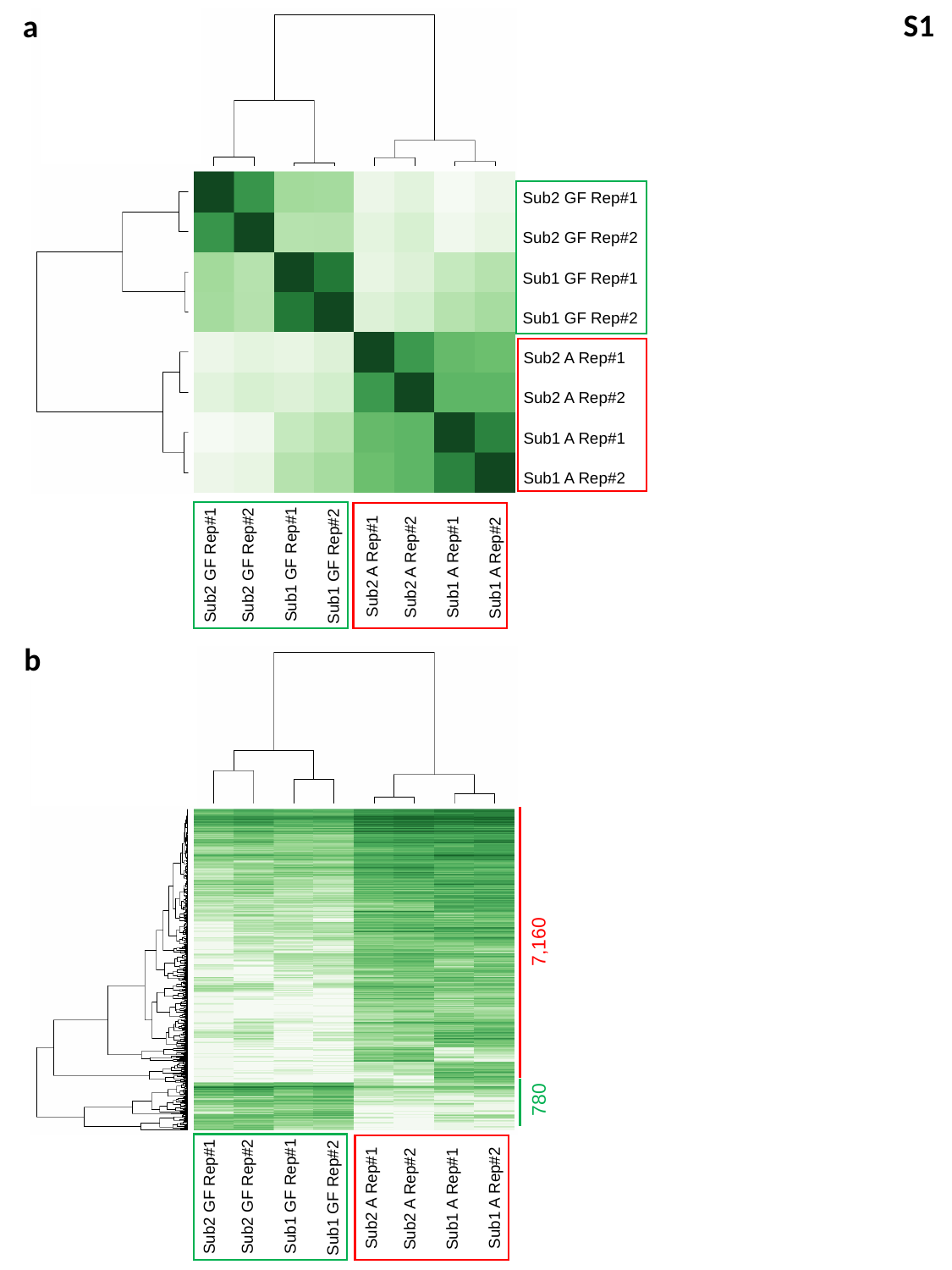

a
S1
Sub2 GF Rep#1
Sub2 GF Rep#2
Sub1 GF Rep#1
Sub1 GF Rep#2
Sub2 A Rep#1
Sub2 A Rep#2
Sub1 A Rep#1
Sub1 A Rep#2
Sub1 GF Rep#1
Sub2 GF Rep#1
Sub2 GF Rep#2
Sub1 GF Rep#2
Sub2 A Rep#1
Sub2 A Rep#2
Sub1 A Rep#1
Sub1 A Rep#2
b
7,160
780
Sub1 GF Rep#1
Sub2 GF Rep#1
Sub2 GF Rep#2
Sub1 A Rep#2
Sub1 GF Rep#2
Sub2 A Rep#1
Sub2 A Rep#2
Sub1 A Rep#1

## Slide 2
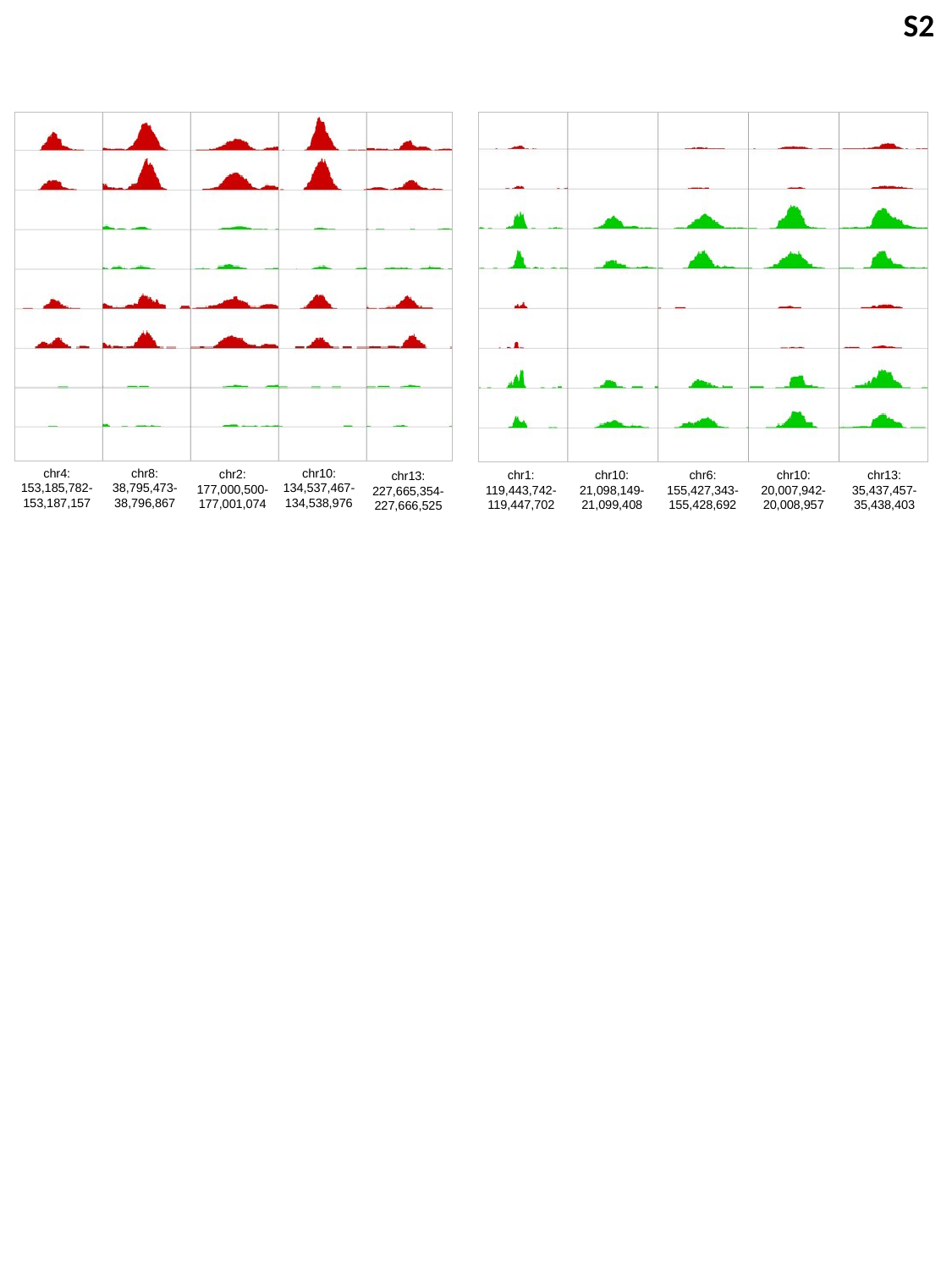

S2
chr8:
38,795,473-
38,796,867
chr4:
153,185,782-
153,187,157
chr10:
134,537,467-
134,538,976
chr2:
177,000,500-
177,001,074
chr13:
227,665,354-
227,666,525
chr1:
119,443,742-119,447,702
chr10:
21,098,149-
21,099,408
chr6:
155,427,343-
155,428,692
chr10:
20,007,942-
20,008,957
chr13:
35,437,457-
35,438,403

## Slide 3
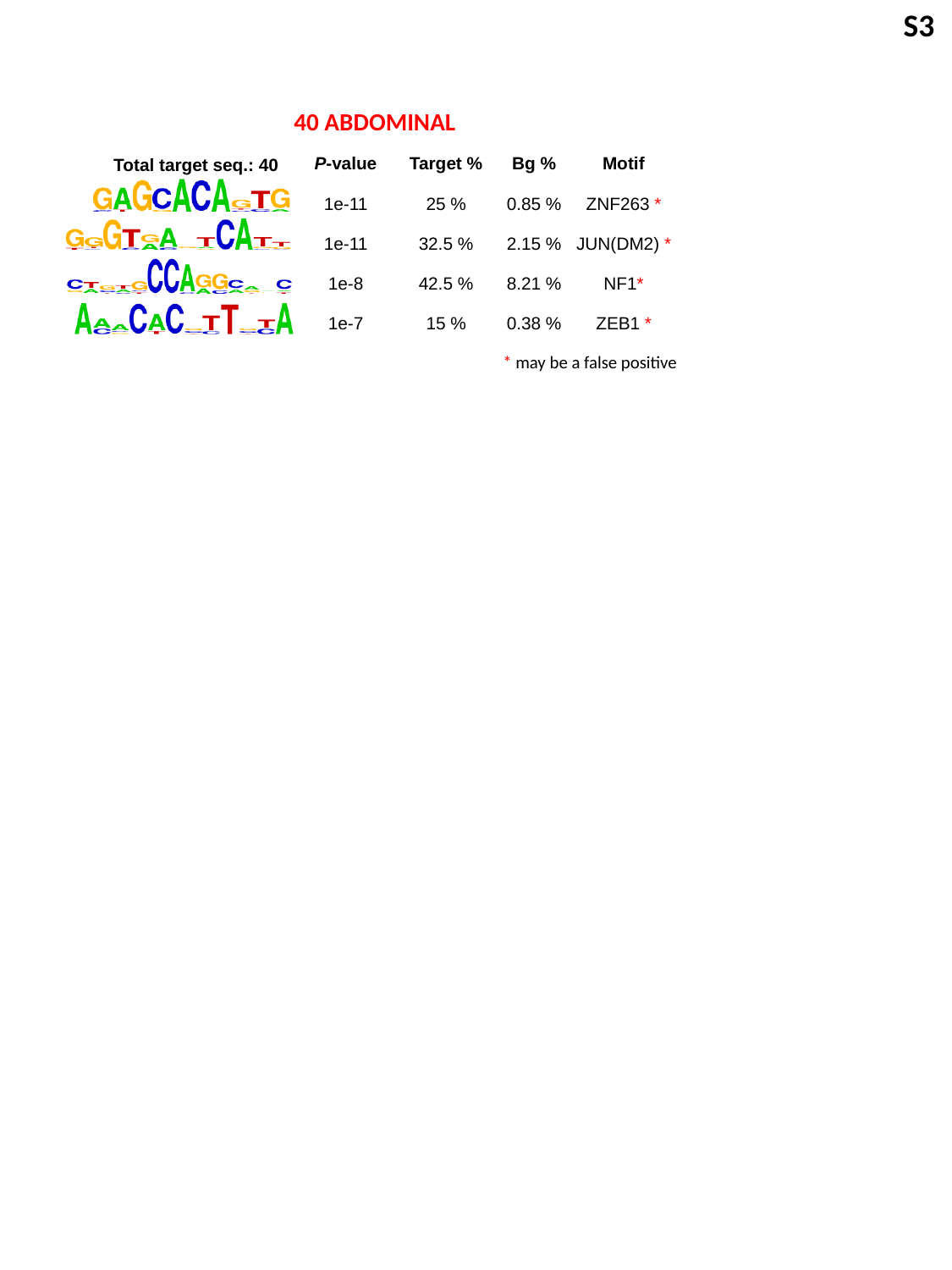

S3
40 ABDOMINAL
| P-value | Target % | Bg % | Motif |
| --- | --- | --- | --- |
| 1e-11 | 25 % | 0.85 % | ZNF263 \* |
| 1e-11 | 32.5 % | 2.15 % | JUN(DM2) \* |
| 1e-8 | 42.5 % | 8.21 % | NF1\* |
| 1e-7 | 15 % | 0.38 % | ZEB1 \* |
Total target seq.: 40
* may be a false positive
